# Investigating the Impact of Acetabular Dysplasia on Sexual Dysfunction and Psychological Well-Being in Women

**DOI:** 10.3390/jcm14072385

**Published:** 2025-03-30

**Authors:** Özgür Ağlamış, Selver Kübra Akkaya, Burcu Erol, Seval Yılmaz Ergani

**Affiliations:** 1 Departmant of Obstetrics and Gynecology, Private Clinic, 34360 Istanbul, Turkey; ozgurztb@gmail.com (Ö.A.); kubrarik@hotmail.com (S.K.A.); 2 Departmant of Obstetrics and Gynecology, Ordu Training and Research Hospital, 52200 Ordu, Turkey; burcusukrany@gmail.com; 3 Department of Perinatology, Etlik City Hospital, 06170 Ankara, Turkey

**Keywords:** acetabular dysplasia, sexual dysfunction, pelvic floor dysfunction, psychological well-being, pain assessment

## Abstract

**Background:** This study aimed to explore the relationship between acetabular dysplasia (AD), a known risk factor for pelvic floor dysfunction (PFD), and its effects on sexual function and psychological well-being in women. **Methods:** A cross-sectional study was conducted with 125 female patients experiencing genitopelvic pain and penetrative disorders. Participants were categorized into AD-positive and AD-negative groups based on anteroposterior pelvic radiographs. Sexual function was evaluated using the Golombok–Rust Sexual Satisfaction Inventory (GRISS), while pain and psychological well-being were assessed using the Visual Analog Scale (VAS) and the Beck Depression Inventory (BDI), respectively. **Results:** Results showed that AD-positive patients had significantly higher pain (VAS: 8.15 ± 1.1, *p* < 0.001) and anxiety scores (BDI: 12.3 ± 11.0, *p* < 0.02). Sexual function was notably impaired in the AD-positive group, with lower sexual satisfaction and a higher prevalence of anorgasmia (*p* = 0.01). The AD-negative group demonstrated better scores in frequency and satisfaction with touch (*p* = 0.04, *p* = 0.03). Additionally, AD-positive patients exhibited limited hip rotation and a higher incidence of osteoarthritis and Legg–Calvé–Perthes disease. **Conclusions:** This study highlights the broader impact of AD on women’s quality of life, emphasizing the need for targeted therapeutic interventions to address sexual dysfunction and psychological distress in affected patients.

## 1. Introduction

Acetabular dysplasia (AD) is a skeletal disorder characterized by insufficient femoral head coverage by the acetabulum [1]. There is a predominance of left-sided (64.0%) and unilateral disease (63.4%). The prevalence in adults varies between 0.1% and 12.8%, and it occurs 2.76-fold more frequently in women than in men [2]. The incidence per 1000 live births ranges from 0.06 in Africans in Africa to 76.1 in Native Americans. There is significant variability in incidence within each racial group by geographic location. The incidence of clinical neonatal hip instability at birth ranges from 0.4 in Africans to 61.7 in Polish Caucasians [3].

Recent studies show that epigenetic factors, such as DNA methylation affect gene expression and therefore could play an important role in developmental dysplasia of the hip pathogenesis [1]. This leads to hip instability and asymmetric stress, initially causing pain and later progressing to osteoarthritis [1]. AD can result from developmental disorders, trauma, infection, vascular impairment (Legg–Calvé–Perthes disease), or iatrogenic factors². Its classical form is linked to genetic and mechanical influences, with intrauterine stress playing a key role in hip misdevelopment [4]. Modern methods also include ultrasonography and 3D assessment by computer tomography (CT) imaging [5]. AD is associated with joint instability and chronic pelvic pain, which may lead to pelvic floor muscle hypertonicity and dysfunction [6].

AD leads to hip joint instability, which increases the mechanical load on the pelvic floor muscles and alters their function. This instability often results in compensatory postural changes, muscle imbalances, and excessive strain on surrounding soft tissues, contributing to pelvic floor dysfunction (PFD). Additionally, altered biomechanics in AD can cause nerve compression, particularly affecting the pudendal nerve, leading to pelvic pain and sexual dysfunction. Studies suggest that correcting AD through surgical interventions improves pelvic stability, alleviating symptoms of PFD and enhancing sexual function. These findings highlight the strong interplay between hip joint biomechanics, pelvic floor health, and overall sexual well-being [5].

Acetabular dysplasia is a chronic condition for which there is no well-defined cure point, and it is an extremely devastating experience. It affects patients’ daily lives and has been shown to have a significant impact on quality of life, particularly in young women. Many DDH patients suffer from chronic hip pain for a long period of time before a correct diagnosis is made and/or intervention is undertaken [7,8]. Insomnia and anxiety exacerbate pain, and over time, economic and social factors interact with physical factors to cause pain and subsequent disability in patients, negatively impacting patients’ psyche [9,10].

Beyond hip pain and degeneration, acetabular dysplasia (AD) is also linked to sexual dysfunction and chronic pelvic pain [11]. AD contributes to sexual dysfunction by causing by pelvic muscle imbalances that develop to stabilize hip movement [12]. This may lead to overactive and painful pelvic floor muscles. PFD manifests through urinary (incontinence, urgency), gastrointestinal (fecal incontinence, constipation), and genital symptoms (pelvic organ prolapse), along with chronic pelvic pain and sexual dysfunction (dyspareunia, orgasmic dysfunction) [13]. AD is known to ameliorate the adverse effects in sexual activity. Limitation of motion was the most common reason for sexual difficulty, which also agreed with previous studies [13]. AD is common among young women and significantly impacts sexual function. Sexual dysfunction affects 30–50% of women in the general population, but its prevalence in PFD patients rises to 83% [14]. Beyond PFD, AD also contributes to sexual dysfunction. Hip joint instability and chronic pain may impair sexual function by limiting movement and causing discomfort during intercourse, leading to dyspareunia and reduced sexual satisfaction [14,15]. The association between AD and sexual dysfunction can be partly attributed to pelvic floor muscle dysfunction, which plays a crucial role in arousal, vaginal lubrication, and orgasmic response [14,16].

The aim of this study is to investigate the association between AD and sexual satisfaction, sexual function, and depressive symptoms in women with vaginismus. For this purpose, we performed our research into a patient group with sexual dysfunction and we evaluated the characteristics of the above dysfunction among patients with different AD status.

## 2. Materials and Methods

This study obtained ethical approval with the reference number HRU/22.17.26 from Harran University Ethics Committee and was conducted between January 2023 and July 2023. After providing informed consent, participants completed self-reported measures of sexual satisfaction and depressive symptoms. Radiographic imaging was performed to assess the presence of AD. Ethical approval was obtained from [name of ethics committee], and this study adhered to the Declaration of Helsinki guidelines.

This study focused on patients who presented to the gynecology clinic with genitopelvic pain and penetration disorders, specifically a subtype of vaginismus, and who were either undergoing treatment or in follow-up after successfully achieving sexual penetration. Patients with a history of pelvic inflammatory disease, tuboovarian abscess, sexually transmitted infections, endometriosis, chronic pelvic pain, degenerative changes in adjacent joints, or those who declined to participate were excluded from this study. A total of 125 patients were included in this study. Patients had not received any physiotherapy treatment, surgery, or hormonal evaluation in the last year. The diagnosis of vaginismus was established based on the patient’s history. Genital examination was performed to rule out any pathologies that could hinder penetration, and routine pelvic X-rays were assessed to evaluate pelvic anatomy. All patients were subjected to the same imaging protocol, an anteroposterior X-ray, with the same equipment, the same staff, and the approval of the same radiologist. Measurement approval was given by the same radiologist, and the measurement was provided by the same orthopedist.

The X-rays were assessed for findings such as osteoarthritis, femoral head osteonecrosis, developmental hip dysplasia, Legg–Calvé–Perthes disease, sequelae of septic arthritis, and dysplastic hip. Those with acetabular dysplasia were categorized as the AD-positive group, while those without it were categorized as the AD-negative group. Orthopedic conditions such as femoroacetabular impingement (FAI), hip osteoarthritis, labral tears, and ligamentum teres injuries were excluded from this study. The supine position was used for flexion, abduction, adduction, internal rotation, and external rotation. The prone position was used for extension. The hip joint range of motion was recorded with the subspinal compression test, in which the affected hip was placed in a hyperflexed position with neutral rotation, the supine position, for flexion, adduction, and internal rotation tests, and the lower limb was placed in a position for flexion, abduction, external rotation tests.

**Figure 1:**
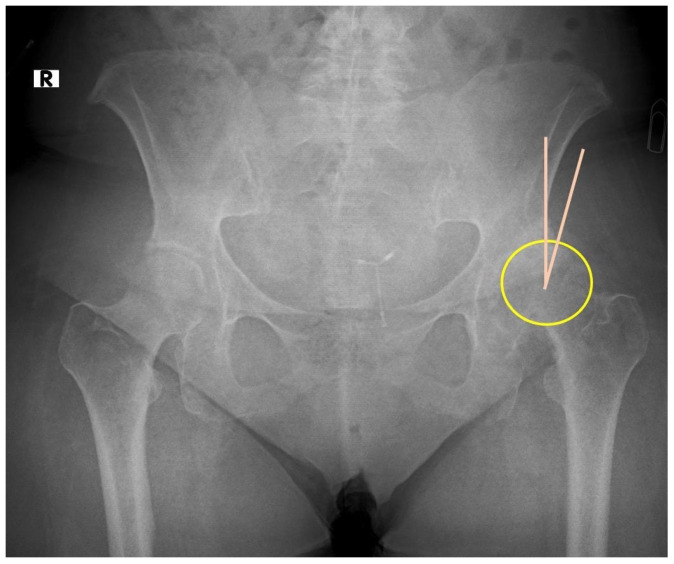
Measured by a vertical line drawn from the center of the femoral head perpendicular to the horizontal reference shown in this figure, CEA is the angle between the center of the femoral head and the lateral edge of the acetabular origin. The angle expressed as α in the figure is 18 degrees (α value of less than 20 degrees is significant for acetabular dysplasia). yellow circle: femoral head; pink line: CEA angle.

**Figure 2:**
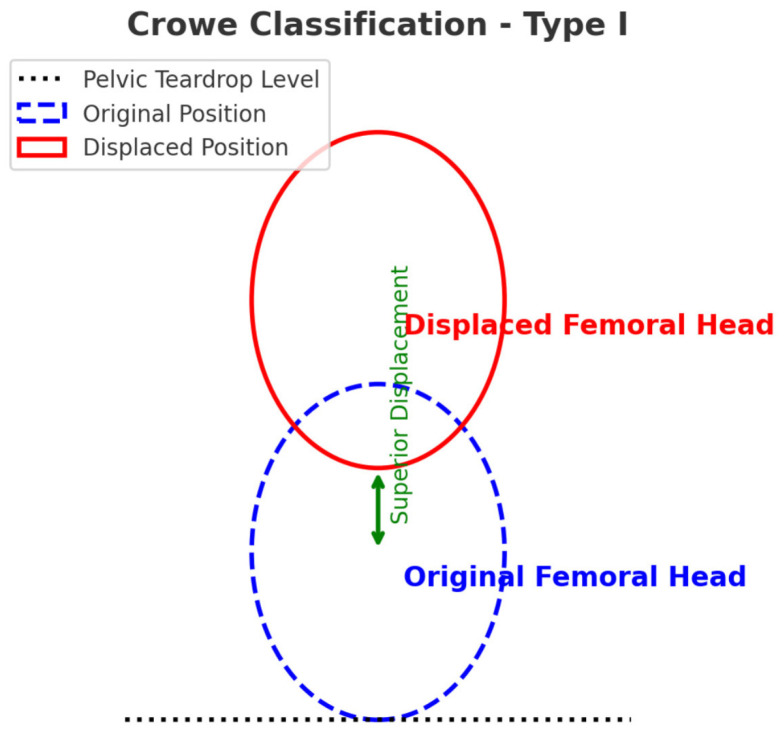
This schematic representation illustrates the superior displacement of the femoral head in a case classified as Crowe Type I. The original position of the femoral head is depicted with a dashed blue outline, while the displaced position is shown in red. The pelvic teardrop level serves as a reference point. In Crowe Type I cases, the superior displacement is less than 50% of the femoral head diameter, indicating mild dysplasia. This classification is crucial for assessing hip joint stability and planning appropriate treatment strategies.

The original position of the femoral head for center edge angle (CEA) analysis was estimated by drawing a vertical reference line from the center of the femoral head perpendicular to the horizontal reference. The center of the femoral head was identified based on insert method, e.g., radiographic landmarks, best-fit circle method, or software-assisted measurement. This ensured a standardized and reproducible assessment of the CEA angle. Traditionally, AD diagnosis relies on anteroposterior pelvic X-rays, where a lateral center-edge angle ≤ 20° is pathognomonic (Figure 1). At the same time, according to Crowe classification [17], the patients were in the Type I category. In a case classified as Crowe Type I, the superior displacement of the femoral head is shown in Figure 2.

In terms of sexual assessment, the Golombok–Rust Sexual Satisfaction Inventory (GRISS) questionnaire [18], consisting of 28 questions, was administered to evaluate sexual function and satisfaction. For the assessment of pain intensity, Visual Analog Scale (VAS) forms were completed by the patients. The GRISS questionnaire divided sexual dysfunction and quality into subcategories, namely, anorgasmia, vaginismus, non-communication, infrequency, female avoidance, female non-sensuality, and female dissatisfaction. It provided Likert-type measurements, ranging ‘never’, ‘rarely’, ‘sometimes’, ‘often’, and ‘always’. Each item was scored between 0 and 4, with some questions reverse-scored. Higher scores indicated worse sexual function and lower sexual satisfaction. After scoring, raw scores were transformed into standardized scores, and a score of 5 or higher indicated a problem in each subcategory. There is no range for scores above 5. Pain scores were measured using the Visual Analog Scale (VAS). The VAS scale is a 10 cm ruler with ‘no pain’ on one end and ‘maximum’ on the other, where patients marked the level of their pain. The patients’ psychiatric status was evaluated using the Beck Depression Inventory (BDI) [19], a 21-item 4-point Likert-type questionnaire used to distinguish anxiety symptoms from depression. The total score ranged from 0 to 63, with higher scores indicating increased anxiety. This highlights the importance of assessing both pain intensity and psychological well-being in patients with acetabular dysplasia.

### 2.1. Power Analysis

A power analysis was conducted to determine the sample size for this study. The sample calculation was performed using G* Power 3.1 software, considering VAS pain scores as the mean. The sample size representing the population was calculated with a significance level of 0.05, a 95% confidence interval, 80% power, and an effect size of 0.5 (medium level). Based on the statistical analysis, it was determined that this study should be conducted with 120 patients. To account for potential dropouts or exclusions, 125 female patients were included in this study.

### 2.2. Statistical Analysis

Data coding and statistical analyses were performed using the IBM SPSS Statistics 22 software package. The normality of the variables was assessed using the Shapiro–Wilk test. The Mann–Whitney U test was used for comparing non-categorical parameters, while chi-square or Fisher’s exact test was used for categorical variables. Pearson and Spearman correlation tests were used for relationships between variables. Multivariate logistic regression analysis was performed. *p* < 0.05 was considered statistically significant.

## 3. Results

Of the 125 participants, 44% were diagnosed with AD (n = 55) and 56% without AD (n = 70). The mean age of the patients in the AD-positive group was 45.58, while in the AD-negative group, it was 46.17. There was no statistically significant difference between the two groups (*p* > 0.05). The ages of the patients ranged between 22 years and 36 years. There was no statistical difference in terms of BMI index of the participants and the mean BMI was 24.7. The average number of births was observed as 2.1, and 75% of the participants had given birth by cesarean section.

Among the findings evaluated in the AP pelvic X-rays, osteoarthritis (41.8%), osteonecrosis of the femoral head (5.4%), developmental hip dysplasia (10.8%), Legg–Calvé–Perthes disease (3.6%), and sequelae of septic arthritis (12.7%) were more frequently observed in the AD-positive patients (Table 1). VAS pain scores (8.15 ± 1.1) and Beck Depression Inventory scores (12.3 ± 11.0) were statistically higher in the AD-positive group (*p* < 0.001, *p* < 0.02) (Table 1). Hip joint movements, including flexion, adduction, and external rotation, were significantly different between the two groups (*p* < 0.001). Flexion and adduction were more limited in the AD-negative group, while external rotation was more restricted in the AD-positive group (Table 2). In the sexual assessment, the GRISS questionnaire revealed that the AD-negative group had better sexual satisfaction (*p* = 0.03) when looking at the total score. When examining the subgroups, frequency and touch were more frequent in the AD-negative group (8.27 ± 1.75) (*p* = 0.04, *p* = 0.03). Anorgasmia was significantly more common in the AD-positive group (7.03 ± 3.68) (*p* = 0.01). Detailed group comparisons for each GRISS subscale are presented in Table 3.

The differences between the AD-positive and AD-negative groups were clearly observed. The mean VAS and BDI scores were significantly higher in the AD-positive group compared to the AD-negative group (*p* < 0.05). Interaction analysis revealed a moderate positive relationship between the severity of acetabular dysplasia and VAS scores (r ≈ 0.4–0.6, *p* < 0.05), indicating that higher severity of AD was associated with increased pain levels. Additionally, a weaker but significant correlation was found between AD severity and BDI scores (r ≈ 0.3–0.5, *p* < 0.05), suggesting that as the severity of AD increases, patients may also experience higher levels of depressive symptoms. In multivariate logistic regression, age is statistically significantly associated with acetabular dysplasia (*p* < 0.05). Since the odds ratio is 4.6, the risk of disease increases with age. BMI is seen to be a significant risk factor. The effect of birth history on acetabular dysplasia is not statistically significant (*p* > 0.05). The OR value being below 1 suggests that this factor may have a protective or weak effect. In patients with osteoarthritis, osteonecrosis of the femoral head, developmental hip dysplasia, Legg–Calvé–Perthes disease, and sequelae of septic arthritis, the risk of developing acetabular dysplasia was found to be statistically significant (Table 4).

## 4. Discussion

In this study, we recruited 125 female patients with a history of sexual dysfunction (genitopelvic pain/penetration disorders) to investigate the differences in sexual dysfunction characteristics and psychological well-being in relation to acetabular dysplasia (AD). Our findings indicate that women with AD exhibit significantly worse sexual function and psychological status. Specifically, standardized assessments using validated tools (BDI score, GRISS questionnaire) revealed that AD-positive women experience more intense anxiety and significant impairments in sexual activity, including lower intercourse frequency, reduced pleasure during touch and caressing, and poorer orgasmic function. These results were independent of age and comorbid pelvic conditions, as patients with known pelvic organ disorders were excluded during recruitment.

High BMI may increase the tendency to acetabular dysplasia by increasing the mechanical load on the joint. Although the effect of BMI on hip dysplasia is controversial in the literature, obesity has been shown to increase the risk of joint diseases. Developmental hip dysplasia was found to be one of the strongest predictors of acetabular dysplasia (*p* = 0.01, OR = 6.5). This finding is consistent with the existing literature that developmental hip dysplasia is a factor that directly leads to acetabular dysplasia. It is known that DDH cases, especially those that are not treated appropriately during childhood, increase the risk of acetabular dysplasia and early osteoarthritis in later ages [20]. Other orthopedic diseases such as osteonecrosis of the femoral head (*p* = 0.02, OR = 2.9), osteoarthritis (*p* = 0.04, OR = 3.8), and Legg–Calvé–Perthes disease (*p* = 0.03, OR = 3.2) were also found to be significant risk factors. It is thought that these diseases may disrupt the biomechanics of the hip joint and lead to acetabular dysplasia. Septic arthritis sequelae were found to be of borderline significance (*p* = 0.05, OR = 4.1), suggesting that the long-term effects of previous infections on the hip joint should be investigated in more detail.

Patients with acetabular dysplasia (AD) may report increased hip flexion and adduction due to biomechanical and compensatory factors associated with hip instability [21]. Since AD is characterized by a shallow acetabulum, it results in decreased femoral head coverage, instability, and altered joint mechanics [22]. Patients often compensate by increasing hip flexion and adduction to maximize contact between the femoral head and acetabulum, thereby improving stability and reducing pain. From an orthopedic perspective, AD-positive patients reported more severe pain and demonstrated altered hip movement patterns, characterized by significantly increased flexion and adduction but restricted external rotation. The higher prevalence of osteoarthritis, septic arthritis, and Legg–Calvé–Perthes disease among AD-positive women was expected, as these conditions are both etiological factors and consequences of AD. Advanced diagnostic methods allow for better assessment of the severity of hip dysplasia. This study highlights its importance. In this way, the effects on social life can be minimized [23]. When the patients were evaluated, it was understood that most of them did not accept the surgery and physiotherapy that were previously recommended. It is obvious that if precautions are taken at a young age, they will recover faster and will not encounter such problems that affect their social lives. Therefore, it is important for AD patients to follow up with the relevant branch and consultation at a young age. Patient education, cognitive behavioral therapy, sex therapy, hypnotherapy, and pelvic floor exercises can be applied as non-surgical treatment options for AD patients with sexual dysfunction [24].

A literature review revealed no direct studies linking AD with female sexual dysfunction. However, indirect evidence suggests that joint-preserving surgical interventions, which aim to prevent further hip degeneration in AD patients, may enhance sexual function [25]. In 2004, Valenzuela et al. found that reorientation surgery (PAO) improved intercourse frequency and satisfaction in 40% and 25% of patients, respectively [26]. Similarly, Masui et al. reported that 68.4% of patients experienced increased satisfaction with sexual activity due to reduced hip pain after PAO [27]. Klit et al. (2014) further confirmed the long-term positive effects of PAO on female sexual quality of life [28]. Additionally, more invasive procedures, such as total hip arthroplasty (THA), have been associated with improvements in sexual function in AD patients [22], as well as in women with other hip conditions [29]. A recent review by Niedenfuehr et al. also emphasized that corrective surgery for AD and other hip disorders enhances sexual activity [30].

When the biological damage experienced by the patient causes a radical negative change in his/her life and seriously impairs his/her quality of life and functionality before it turns into a psychiatric condition, it is considered pathological according to the bio-psycho-social paradigm [31]. Regarding the psychological impact of hip disease, Nisar et al. reported that 32% and 49% of patients with hip disorders exhibit borderline-to-abnormal levels of depression and anxiety, respectively [32]. Additionally, AD patients appear to suffer from poor sleep quality due to worsening nocturnal pain, even before the onset of hip degeneration [33]. These findings align with our study, suggesting that AD comprises a major orthopedic condition, which foremostly impairs the myoskeletal function, and subsequently can evolve into a debilitating factor for the psychosocial dimension of patient life. These effects also compromise the sexual life of the patient and lead to a constellation of disorders with a negative impact on overall life quality. The most common problem during sexual intercourse was the fear of dislocation, followed by weakness in muscle strength. The common reason given was a limitation of motion. Difficulty in leg positioning in female patients affects their quality of life in this regard.

Although AD causes sexual dysfunction, it does not cause problems with vaginal birth. AD is not associated with high-risk complications during pregnancy or with increased difficulty in vaginal delivery [2]. The fact that it does not cause any problems in terms of normal birth may be shown as the reason why these patients are less likely to consult a doctor for sexual dysfunction.

Acetabular dysplasia affects sexual function and psychological well-being by causing hip instability, chronic pain, and movement limitations. Studies show that AD is associated with dyspareunia, reduced sexual satisfaction, and decreased flexibility during intercourse [30]. Pelvic floor dysfunction (PFD) due to altered hip biomechanics may contribute to urinary incontinence and sexual dysfunction [34]. Chronic pain in AD can lead to psychological distress, increasing anxiety and depression, which further exacerbate sexual dysfunction [35]. A study comparing women with and without AD found significantly lower sexual satisfaction and higher depression scores in those with AD [7]. Surgical interventions, such as periacetabular osteotomy, have been shown to improve hip function and alleviate pain, subsequently enhancing sexual health [36,37]. Psychological factors, including fear of pain and avoidance behavior, can negatively impact intimacy and overall quality of life [36]. Addressing both the orthopedic and psychological aspects of AD is crucial for improving patient outcomes. Integrating pelvic floor therapy and mental health support may enhance treatment efficacy. A multidisciplinary approach is recommended to optimize function and quality of life in women with AD.

Radiography is a commonly used method for diagnosing acetabular dysplasia (AD); however, measurement techniques and potential diagnostic errors must be considered. Small variations in patient positioning during radiographic imaging can lead to inaccuracies in assessing acetabular coverage angles [30]. Alternative imaging modalities, such as magnetic resonance imaging (MRI) and computed tomography (CT), improve diagnostic accuracy by providing better visualization of both bony structures and soft tissues [38]. MRI is particularly useful for evaluating labral and cartilage pathologies, while CT enables detailed three-dimensional analysis of acetabular morphology [7]. However, static imaging techniques do not provide functional assessments of joint movement [36]. Recently, dynamic assessment techniques such as vibroarthrography have gained attention for their ability to analyze joint function in real time. Vibroarthrography detects acoustic signals generated by joint movement, allowing for early identification of cartilage degeneration and osteoarthritis progression [35]. Studies suggest that integrating vibroarthrography with conventional imaging modalities could enhance diagnostic precision and provide a more comprehensive understanding of joint health [38]. Future research should explore the clinical applications of vibroarthrography in patients with AD, particularly its potential to guide treatment decisions and predict disease progression [36]. A multimodal imaging approach combining static and dynamic techniques may optimize the evaluation of AD and its impact on pelvic floor dysfunction and sexual health. In this respect, EEMD-DFA algorithms and ANN classification can be used for the detection of knee osteoarthritis using vibroarthrography [39].

## 5. Limitations and Future Plans

While this study provides valuable insights, several limitations should be noted. The cross-sectional design precludes causal inferences, and the reliance on self-reported measures may introduce reporting biases. Additionally, this study focused on a single orthopedic condition, so the findings cannot be generalized to other forms of PFD and to all women.

Limitations of our study include the fact that sexual function was not assessed comparatively after surgical intervention or conservative intervention, and that psychological or sexual therapy experiences did not affect the results. There was a limited number of patients in our article. The fact that this study is not generalizable to all ADs may be considered a limitation. New studies could be conducted to determine the extent of psychological and sexual distress in terms of factors such as body image, perception of sexuality, and awareness of medical conditions. We believe that this article will be an example for prospective or interventional studies to be conducted in the future.

## 6. Conclusions

This study is the first known study in the literature to investigate the differences in sexual function, psychological well-being, and pain indices between women with and without AD. Acetabular dysplasia significantly affects sexual satisfaction and depressive symptoms in women with vaginismus. Integrating a multidisciplinary approach that takes into account both orthopedic and psychological aspects in the management of patients with acetabular dysplasia, and questioning sexual life, which is not considered at first glance but affects the patient’s quality of life quite negatively, will positively affect the social, physical, sexual, and psychological lives of patients. In this sense, quality of life questionnaires, sexual satisfaction questionnaires, and depression questionnaires can be routinely used to understand whether such patients are affected psychologically. We believe that this study will be an example for future studies. In this context, we need more data and studies that evaluate this patient group not only from an orthopedic perspective but also from a gynecological, psychiatric, and psychological perspective.

## Figures and Tables

**Table 1:** Pelvis anterior posterior radiography evaluation.

	AD Positive (n = 55)	AD Negative (n = 70)	*p*
Age, mean, SD (years)	45.58 ± 14.09	46.17 ± 14.25	0.72
Osteoarthritis, n (%)	23 (41.8)	13 (18.5)	**0.012**
Osteonecrosis of the femoral head	3 (5.4)	2 (2.8)	0.116
Developmental hip dysplasia	6 (10.8)	3 (4.2)	0.102
Legg–Calvé–Perthes disease	2 (3.6)	1 (1.4)	0.385
Sequelae of septic arthritis	7 (12.7)	2 (2.8)	**0.014**
VAS score, mean ± SD	8.15 ± 1.1	1.26 ± 1.06	**<0.001**
Beck Depression Inventory Score ± SD	12.3 ± 11.0	6.1 ± 6.7	**0.02**

AD: acetabular dysplasia; SD: standard deviation; VAS: Visual Analog Scale. Mann–Whitney U and Student *t* tests were used for numerical variables. Data are given as mean ± standard deviation, n (%), median (min–max). Results were accepted at 95% confidence interval and *p* value ˂ 0.05 significance.

**Table 2:** Range of motion of the hip joint.

	AD Positive Mean ± SD (n = 55)	AD Negative Mean ± SD (n = 70)	*p*
Extension	15.6 ± 4.6	16.9 ± 6.8	0.2
Flexion	137.1 ± 8.4	119.7 ± 7.9	**<0.001**
Abduction	44.1 ± 5.01	44.25 ± 5.3	0.9
Adduction	13.1 ± 2.8	10.3 ± 3.8	**<0.001**
Internal rotation	49.8 ± 7.7	45.8 ± 6.1	0.1
External rotation	47.8 ± 6.9	50.25 ± 12.75	**<0.001**

AD: acetabular dysplasia; SD: standard deviation. Chi-square test was performed. Data are given as mean ± standard deviation, n (%), median (min–max). Results were accepted at 95% confidence interval and *p* value ˂ 0.05 significance.

**Table 3:** Evaluation of The Golombok–Rust Inventory of Sexual Satisfaction (GRISS) according to groups.

	AD Positive Mean ± SD (n = 55)	AD Negative Mean ± SD (n = 70)	*p*
Frequency	3.3 ± 1.5	4.01 ± 1.8	**0.04**
Communication	2.76 ± 2.1	2.54 ± 1.9	0.46
Satisfaction	6.15 ± 2.08	6.75 ± 2.71	0.32
Avoidance	5.15 ± 4.16	4.32 ± 3.62	0.21
Touch	7.55 ± 1.70	8.27 ± 1.75	**0.03**
Vaginismus	6.03 ± 3.36	5.42 ± 2.76	0.2
Anorgasmia	7.03 ± 3.68	5.15 ± 2.86	**0.01**
Total	37.97 ± 18,58	36.56 ± 17.4	**0.03**

AD: acetabular dysplasia; SD: standard deviation. Fisher’s exact test was performed. Data are given as mean ± standard deviation, n (%), median (min–max). Results were accepted at 95% confidence interval and *p* value ˂ 0.05 significance.

**Table 4:** Logistic regression analysis of parameters for predicting acetabular dysplasia.

	B	OR	95% CI	*p*
Age	0.18 ± 0.04	4.6	0.1 ± 0.2	0.01
BMI	0.35 ± 0.06	5.2	0.2 ± 0.4	0.01
Birth history	−6.15 ± 2.08	0.3	−0.5 ± 0.4	0.76
Osteoarthritis	0.45 ± 0.08	**3.8**	0.3 ± 0.5	**0.04**
Osteonecrosis of the femoral head	0.62 ± 0.09	**2.9**	0.4 ± 0.6	**0.02**
Developmental hip dysplasia	1.10 ± 0.12	**6.5**	0.8 ± 1.2	**0.01**
Legg–Calvé–Perthes disease	0.75 ± 0.10	**3.2**	0.5 ± 0.9	**0.03**
Sequelae of septic arthritis	0.92 ± 0.11	**4.1**	0.7 ± 1.0	**0.05**

LR: logistic regression; OR: odds ratio; CI: confidence interval. Multivariate logistic regression was performed. Data are given as mean ± standard deviation. Results were accepted at 95% confidence interval and *p* value < 0.05 significance.

## Data Availability

The datasets used and/or analyzed during the current study are available from the corresponding author upon reasonable request.
